# Reducing carbon emissions in the cement industry using effective measures based on countries’ characteristics

**DOI:** 10.1371/journal.pone.0311859

**Published:** 2024-11-21

**Authors:** Hui Gao, Donglin Wang, Zhongwei Zhao, Pei Dang

**Affiliations:** 1 School of Civil Engineering, Liaoning Technical University, Fuxin, China; 2 School of Economics and Management, Tianjin Chengjian University, Tianjin, China; University of the West of England, UNITED KINGDOM OF GREAT BRITAIN AND NORTHERN IRELAND

## Abstract

Cement production contributes 5% of global anthropogenic CO_2_ emissions (CEs), and more than 90% of the CEs are in the procedure of pyroprocessing. Thus calculating the pyroprocessing CO_2_ emission number (PCEN), determining CE-impacted factors, and investigating tailored measures of PCEN reduction for countries based on their characteristics is quite necessary. More specifically, different countries can meet different obstacles to reducing PCENs, such as different restrictions on natural resources and policies, improper energy structures, and so on. With this in mind, tailored measures for PCEN reduction in different countries should be investigated and developed based on their own characteristics. This study selects four sample countries, China, the US, Australia, and Turkey, because of their representative locations and characteristics and then determines PCENs for these countries. The results show that the PCENs of China, the US, Australia, and Turkey are 884, 886, 828, and 913 kgCO_2_/t clinker, respectively. Subsequently, the most PCEN-impacted factors are analyzed, and the reasons for sample countries’ different PCENs are discussed. Then, corresponding custom measures are proposed for each country based on its characteristics. The measures proposed in this study can help with the PCE reduction in the world’s cement industry, and the collected data and calculated results can be used to further research on improving energy conservation and emission reduction measures.

## 1. Introduction

The greenhouse effect has been a serious problem because of increasing developments in industry and technology in the 20th century all over the world. From 1972 to 2011, global GHG emissions, which are usually simplified as CO_2_ emissions (CEs), increased from 14.8 to 31.3 Gt, 63% of which are contributed by industrial activities, showing a significant impact on climate change [1]. For instance, the maximum temperature of the Arctic Circle in July 2022 reached an unprecedented 32.5°C, which can cause billions of tons of ice-out to elevate the global sea level, and this serious incident has been noticed around the world. Also, the fast ice off Utqiaġvik (Alaska) disappeared rapidly because of the high temperature, which caused significant ecological problems in the place. The CEs are mostly caused by the combustion of huge quantities of carbon-intensive fossil fuels, such as coal and natural gas, to generate the required power for industries. Moreover, some industrial processes have reactions that chemically generate CO_2_, such as the pyroprocessing procedure of cement production which is considered as one of the most important greenhouse pollution contributors worldwide (accounting for 5% of global anthropogenic CEs) [2–4]. More seriously, according to Gursel’s research [5], the world cement demand and production are predicted to grow from 2540 million tonnes (Mt) in 2006 to around 3,680–4,380 Mt in 2050. The largest share of this growth will take place in developing countries, such as China and India. Obviously, the increase in demand and production will result in a considerable increase in the cement industry’s CEs in the future.

As the largest producer of cement, China produced 58% of the world’s cement (2.48 billion metric tons) in 2014, growing by 150% since 2004, and has been the world’s largest CO_2_ emitter in the cement industry [6, 7]. After reaching its peak in 2014, China’s cement production slightly reduced to 2.21 billion metric tons in 2018 and then recovered to 2.36 billion metric tons in 2021 [7]. After the recent years’ slow economic growth because of the well-known disease disaster in the world, China’s cement requirements and production are predicted to grow in the short future because of the continuous modernization and economic bounceback, having been the obstacle to China’s strategic goal of reaching a carbon peak by 2030 and carbon neutrality by 2060 (China’s two-carbon goal) and giving an urgent requirement to generate measures to reduce CEs in the cement industry. Previously, scholars have proposed studies of CEs in China’s cement industry. Hu et al. [8] and Shen et al. [9] developed emission factors in their study to evaluate pollutant emissions from different types and scales of clinker and cement production lines. In the meanwhile, the life-cycle environmental impact of certain cement production lines and China’s cement industry was analyzed by Song et al. [10], Li et al. [11], Chen et al. [12], and Shen et al. [13], respectively. Moreover, Gao et al. [14] and Shan et al. [15] estimated both direct and indirect CEs for China’s cement industry, and the CEs due to cement production in China for a continuous period from 1980 to 2014 is especially analyzed by Gao et al. [14]. In addition, Zheng et al. [16] further estimated the direct and indirect emissions of GHGs for China’s cement industry from 2005 to 2012 at the provincial level from the perspective of the cement life-cycle and stated that the comprehensive energy consumption per cement product was reduced during this period. Unfortunately, some of the current CE reduction measures for the cement industry are outdated or not appropriate to be sustainably applied. For instance, according to the National Bureau of Statistics [17], the comprehensive energy consumption per cement product reduced from 110 to 80 tce/kg during 2005–2012, which is mainly because of replacing the traditional shaft kilns with advanced preheater kilns [14, 16]; however, the effect of further application of this measure can be quite limited for China as the percentage of preheater kilns reached 92% in 2012, meaning that there is not much space for kiln improvement to further reduction of CEs before the new kiln technology leap. Thus, looking abroad to look for successful examples and generate new measures based on China’s own characteristics to sustainably reduce CO_2_ emissions for the cement industry becomes quite necessary.

Raise the horizon to the world, despite the rapid growth of cement production in developing countries due to increasing demand for the necessary housing and infrastructure, a noticeable decrease in cement production appears in the United States and the EU. For the US, cement manufacturing decreased significantly at a rate of 21% from 2004 to 2013 with a production share of only 1.9% of the world’s cement production, which is very close to Turkey’s contribution at a level of 1.8% [5]. EU also experienced a significant decrease in cement production from 2004 to 2011, resulting in the concrete output decreasing from 355.8 to 269.9 million m^3^ [18]. However, the longer-term forecast for worldwide cement and concrete manufacturing stays positive because of the contribution from developing countries, which requires more measures to reduce CEs from cement production. Previously, Hasanbeigi et al. [19] described 18 emerging energy efficiency options and CE reduction technologies for cement production. Bosoaga et al. [20], Worrell [21], Ogbeide [22], Stocks et al. [23], and Stefanovic et al. [24] analyzed the effectiveness of several techniques for reducing CEs in the cement manufacturing process. Hasanbeigi [25] compared the average primary energy of 16 surveyed cement plants with the international best practice levels and found that the average technical potential primary energy can save 23% if the plants operated at international best practice levels. Additionally, Çankaya and Pekey [3], Gursel [5], Canpolat [26], and Thwe et al. [27] investigated the CEs of the cement industry in Turkey and Myanmar, respectively, and analyzed techniques to reduce CEs. Visintin et al. [28], Shobeiri et al. [29], Gravina et al. [30], Gravina and Xie [31], and Bennett et al. [32] collected CE-related data in Australia and proposed emission factors combined with the mechanical and durability-related properties to analyze the relationship between CE and mechanical and durability-related properties of the products. In summary, the previous research more or less gave measures to reduce CEs for cement and concrete products, but the views of most of the previous studies are limited to one country or region, and the proposed calculation models and CE reduction measures might be used for one country only and cannot be promoted to other regions.

Cement production includes several procedures, such as quarrying, transportation, pyroprocessing, clinker cooling, finish milling, grinding and bending, and others. However, pyroprocessing consumes around 88% of the total fuel and 91% of the total energy used in all the cement production procedures and is responsible for CEs in the same proportion among all the procedures [5, 33]. Thus, focusing on reducing CO_2_ emissions in pyroprocessing instead of the whole cement production process can be more concise, efficient, and effective. Most of the previous studies focus on all the cement procedures or even concrete production procedures, which makes the studies too complicated to develop targeted measures to reduce CEs for cement production. Moreover, most of the previous studies generally focus on one country or area instead of a global vision, but the CE reduction measures that are effective in one country may not be useful for other countries, and the PCENs of countries which are defined as the mass of CO_2_ emitted in producing one tonne of clinker in pyroprocessing may not all be recorded in literature. Besides, mutual learning between countries on different continents can be helpful to further reduce PCEs globally. Furthermore, the methods for reducing PCEs should synthetically take all advantages and limitations into consideration for the specific country, which is not paid much attention to by most of the previous studies. Therefore, calculating the PCENs of countries and estimating the CE impact factors are insufficient and desired, and a comprehensive provision of effective and efficient CE reduction measures for the cement industry in different countries and regions based on their characteristics is also urgently required.

**Fig 1:**
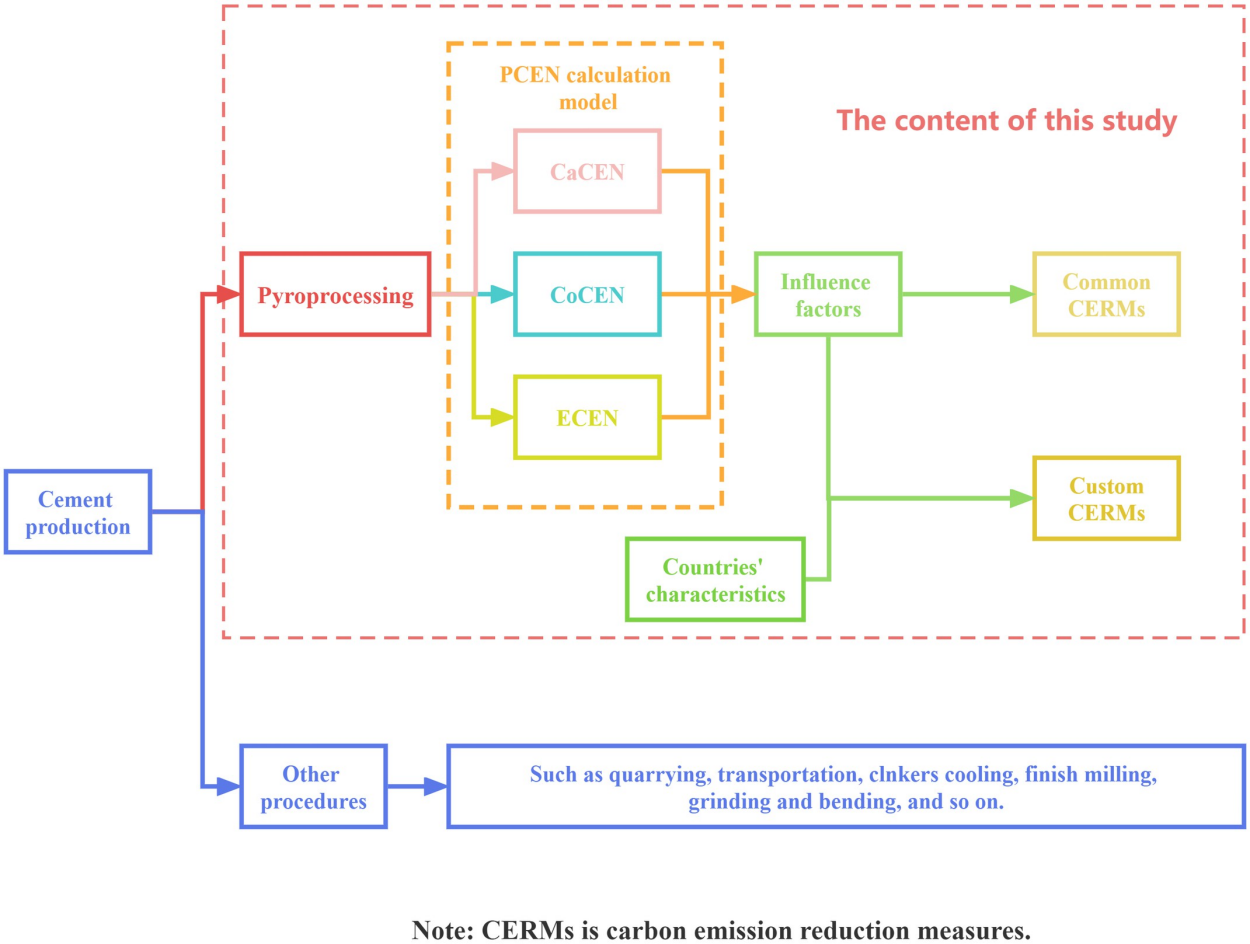
The research framework of this study.

According to these research gaps, the aim of this study is to calculate pyroprocessing CO_2_ emission numbers (PCENs) for the selected sample countries and evaluate the impactful factors of CEs. More importantly, specific measures are provided for reducing PCEs in certain countries based on their characteristics reasonably and effectively. Accordingly, Fig 1 illustrates a brief vision of the framework of this study. The first objective of this study is to propose an analytical model that can be used for all countries to calculate the PCENs. The second objective is to collect data from the most related publications and authoritative resources, such as the National Bureau of Statistics and high-cited papers, to calculate the PCENs for four selected sample countries, The US, China, Australia, and Turkey. The subsequent objective is to identify factors that influence the PCEN during pyroprocessing, give common cement CE reduction measures, and analyze reasons for different PCENs of sample countries. The fourth objective is to discuss the PCE reduction potential of the sample countries and specifically give custom PCE reduction measures. The last objective of this study is especially to give specific measures for China because of its largest cement production in the world and the severe environmental situations that the country is facing. This study focuses on pyroprocessing-related CO_2_ emissions, while other types of pollutants, such as PM 2.5 and NO_x_, are not included. Other related procedures in the cement industry are concisely mentioned but not analyzed and discussed. The collected data and analytical results given in this study can help engineers and researchers to further investigate techniques for CE reductions and provide policymakers the suggestions to further improve the energy conservation and emission reduction policies for cement-related industries in different countries. It can be predicted that the measures proposed in this work will facilitate the more effective and efficient reduction of CEs in real-world cement industrial practice for different regions and countries based on their own characteristics.

## 2. Analytical model for calculating PCEN

Pyroprocessing is considered to be responsible for more than 90% of the total energy used and CO_2_ emitted in cement production [5], so focusing on the CO_2_ emissions in pyroprocessing instead of the whole cement production process can be more concise, effective, and efficient. In this study, the GHG emission is simplified as CO_2_ emission, since for most fuels the difference between the GHG emission factor and CO_2_ emission factor is less than 1% [5, 14, 34]. The PCE is from three sources: calcination of calcium carbonates, fuel combustion, and electricity consumption. Thus, the PCEN can be calculated by summing the calcination CO_2_ emission number (CaCEN), the combustion CO_2_ emission number (CoCEN), and the electricity CO_2_ emission number (ECEN), as shown in Eq (1).

$$E_{p}=E_{Cal}+E_{Com}+E_{Elec}$$ (1)

where *E*_*P*_ is the PCEN; *E*_*Cal*_ is the CaCEN; *E*_*com*_ is the CoCEN; and *E*_*Elec*_ is the ECEN.

It should be noted that the clinker-to-cement ratio can significantly influence the CO_2_ emission factor (CEF) of cement. For instance, Liu et al. [35] avoided the use of default clinker-to-cement ratios (75% and 95% in the IPCC guidelines 1996 and 2006, respectively), resulting in the obtained CO_2_ emission factors (CEFs) of cement being 32%–45% lower than the IPCC ones. This is because the clinker-to-cement ratio used by Liu et al. [35] is 23% lower than the latest IPCC default values. Therefore, in this study, PCEN, CaCEN, CoCEN, and ECEN refer to clinker instead of cement. After PCEN is obtained, the cement CEF can be calculated easily based on the clinker-to-cement ratio.

### 2.1 Calcination CO_2_ emissions

The calcination (chemical) CO_2_ is emitted during a series of chemical reactions which convert calcium and silicon oxides into calcium silicates in the kilns. When clinker is produced from raw materials, the calcination process of calcium carbonate (CaCO_3_) releases CO_2_. The reaction equation When the temperature in the kiln reaches about 900°C, the limestone (main component is calcium carbonate) undergoes a chemical reaction which can be called calcination, and calcium oxide is formed with the emission of CO_2_. is CaCO_3_→CaO + CO_2_ [34]. Based on the chemical reaction, Liu et al. [35] proposed a solid method to use molar mass to calculate the CaCEN according to the principle of element balance, and the equation was expressed as:

$$E_{Cal}=E_{CaO}\times (1+R_{D})$$ (2a)

and

$$E_{CaO}=F_{CaO}\times \frac{{Mole}_{{CO}_{2}}}{{Mole}_{CaO}}$$ (2b)

where *E*_*CaO*_ is the mass of total CO_2_ emission released as CaO per unit of clinker (kgCO_2_/t clinker); *R*_*D*_ is the cement kiln dust (CKD) correction factor (%); *F*_*CaO*_ is the mass proportion of CaO per unit clinker (%); ${Mole}_{{CO}_{2}}$ is the mole mass of CO_2_ (44); and *Mole*_*CaO*_ is the mole mass of CaO (55.68). It should be noted that *R*_*D*_ can be zero if the CKD is not considered for a simple calculation.

### 2.2 Combustion CO_2_ emissions

The combustion CO_2_ emissions are caused by burning fuels in the kilns and can be calculated from the combustion data. Thus, the equation for calculating the CoCEN is as follows:

$$E_{Com}=\sum_{i=1}^{n}E_{Comi}=\sum_{i=1}^{n}\frac{{Energy}_{T}\;\times Mass\;\times Proportion\;}{{HHV}_{i}}\;\times E_{{iCO}_{2}}$$ (3)

where *E*_*Comi*_ is the CO_2_ emission per unit mass of clinker for fuel *i*; *Energy*_*T*_ is the average (kiln) thermal energy consumption for producing 1 unit mass of clinker in different types of kilns; *Mass* is the mass of produced clinker and here is set to be 1 kg; *HHV*_*i*_ is the high heating value of fuel *i*; and $E_{{iCO}_{2}}$ is the CO_2_ emitted from combusting 1 unit mass of fuel *i*.

### 2.3 Electricity CO_2_ emissions

The electricity CO_2_ emission number (E_Elec_) is caused by the electricity consumption in pyroprocessing, which can be separated into two parts: the electricity used for operating the kiln, and for milling and drying the solid fuels (fuel preparation), such as coal and coke. The total consumed electricity is generated in the electricity plants by consuming different types of fuels, emitting CO_2_. Thus, the ECEN can be calculated as follows:

$$E_{Elec}={(Electricity}_{K}+\;\sum_{i=1}^{n}{\;Fuel}_{\%}\times {ECFKFP}_{i})\times Mass\times E_{{CO}_{2}}$$ (4)

where *E*_*Elec*_ is the electricity CO_2_ emission number; *Electricity*_*K*_ is the electricity used by the kiln; *Fuel*_*%*_ is the proportion of pyroprocessing used solid fuel *i*; *ECFKFP*_*i*_ is the electricity consumption factor for solid kiln fuel *i* preparation; *Mass* is the mass of produced clinker; and $E_{{CO}_{2}}$ is the electricity-generation CO_2_ emission factor.

## 3. Data collection and PCEN calculation for sample countries

In this section, the data collected by the authors and the corresponding calculation results of PCENs for the four selected representative countries: China, the US, Australia, and Turkey are presented. Among these countries, China has more than half of the world’s cement production; the US is the most developed country; Australia has performed very well in environmental protection and locates in the southern hemisphere; Turkey is experiencing rapid development in cement production and locates in the Middle East. Thus these four countries are representative of a global view of the world’s cement industry. The collected data and calculated results in this section can be used to find the advantages and disadvantages of each country, and then to purposefully give strategies to each country for reducing CO_2_ emissions in pyroprocessing.

### 3.1 CaCEN estimation

The CaCEN can be slightly different for different countries, but the difference is quite small as this chemical reaction is stable and follows the principle of element balance [5, 35]. Clinker is the major CO_2_ emitter element of cement, but data on clinker production is less widely reported than that on cement production, resulting in data about clinker production are not available [35]. Thus, *F*_*CaO*_ (and *R*_*D*_) can vary for different authoritative literature, resulting in different calculation results of CaCENs [14, 35, 36]. For the US, the CaCEN (E_cal_) was estimated as 522kg/t clinker [5, 37]. For China, the CaCENs were calculated by some authoritative literature, such as IPCC [38] (507.27 kgCO_2_/t clinker), Gao et al. [14, 38] (521 kg/t clinker), Liu et al. [35] 495 kgCO_2_/t clinker), Zheng et al. [16] (550 kgCO_2_/t clinker), Lei et al. [39] (550 kgCO_2_/t clinker), and NDRC [40] (501 kgCO_2_/t clinker). It can be seen that the calculation results are relatively different, but the difference is within ± 10%. This study adopts the calculation result of Gao et al. [14, 36] (521 kgCO_2_/t clinker) as it is closest to the median, which is also in consistent with Geng et al. [41] (520 kgCO_2_/t clinker), and also according to the CaCEN of the US as explained in the beginning of this paragraph [5, 35]. The calcination CO_2_ numbers of Australia and Turkey are provided in Bennett et al. [32] and Gursel [5] as 522 kgCO_2_/t clinker.

### 3.2 CoCEN calculation

The combustion CO_2_ emissions are caused by burning fuels in the kilns and can be calculated from the combustion-related data. Table 1 lists the proportions of the fuel mixes used in pyroprocessing for the four countries. Table 2 gives the combustion data of the fuel mixes listed in Table 1. It should be noted that coal is nearly the total fuel used in China’s clinker production, and the share of alternative fuels can be negligible [14, 42]. Moreover, the coal used in China is traditionally referred to as raw coal [42], and this name is consistently used in this study. Similarly, the waste fuel used in the US and Australia is constituted by waste oil, waste tire, waste solvent, waste paper, and others, and thus is referred to as waste (total) for brevity. The average (kiln) thermal energy consumption for producing 1 unit mass of clinker in different types of kilns (*Energy*_*T*_) is given in Table 3, and *HHV*_*i*_ and $E_{{iCO}_{2}}$ can be seen in Table 2. Thus, according to Eq (3), the calculated CoCENs (kgCO_2_/t clinker) are 294, 329, 274, and 342 kg/t clinker for China, the US, Australia, and Turkey, respectively.

**Table 1:** The proportions of pyroprocessing used fuel types in the four countries [5, 14, 32, 42].

Kiln fuels	by energy, China (%)	by energy, US (%)	by energy, Turkey (%)	by energy, AU (%)
**Raw coal**	100	\	\	\
**Bituminous coal**	\	64	27	57
**Lignite coal**	\	\	31	\
**Petroleum Coke**	\	21	43	1
**Natural gas**	\	4	\	34
**Waste (total)**	\	11	\	7
**Distillate oil**	\	1	\	1

**Table 2:** Combustion data of the fuels [14, 43–49].

	Unit	Raw coal	Bituminous coal	Lignite coal	Petroleum coke	Natural gas	Waste (average)^*a*^
Unit		kg	kg	kg	kg	m^3^	kg
**HHV (Higher Heating Value)**	(MJ per unit)	29.3	27.91	15.12	35.7	38.23	30.1
**CO**_**2**_ **per HHV of fuel**	kg/MJ	84	94.9	196	88.9	51	72
**CO**_**2-eq**_	kg	\	2.66	2.47	3.68	1.94	2.53
**CO**_**2**_	kg	2.46	2.65	2.45	3.59	1.94	2.51

**Table 3:** Pyroprocessing thermal energy consumption for different kilns in the US and China [5, 33, 50].

Thermal Energy Consumption (MJ/kg Clinker)			
Technology Options	Avg	Max	Min
**Wet Kiln**	6.2	6.9	5.4
**Shaft Kiln**	5.2	\	\
**Long dry kiln**	5.0	5.4	4.6
**Preheater kiln**	3.5	3.8	3.1
**Preheater/precalciner kiln**	3.3	3.6	3
**US Average kiln**	3.5	3.8	3.1
**China Average kiln** ^a^	3.5	\	\

^*a*:^Estimited by the authors based on data collected from [14, 16], which is in consistent with Georgiades et al. [51] (3.39 MJ/kg Clinker) and Sousa [52] (between 3.4 and 3.65 MJ/kg Clinker).

### 3.3 ECEN calculation

**Fig 2:**
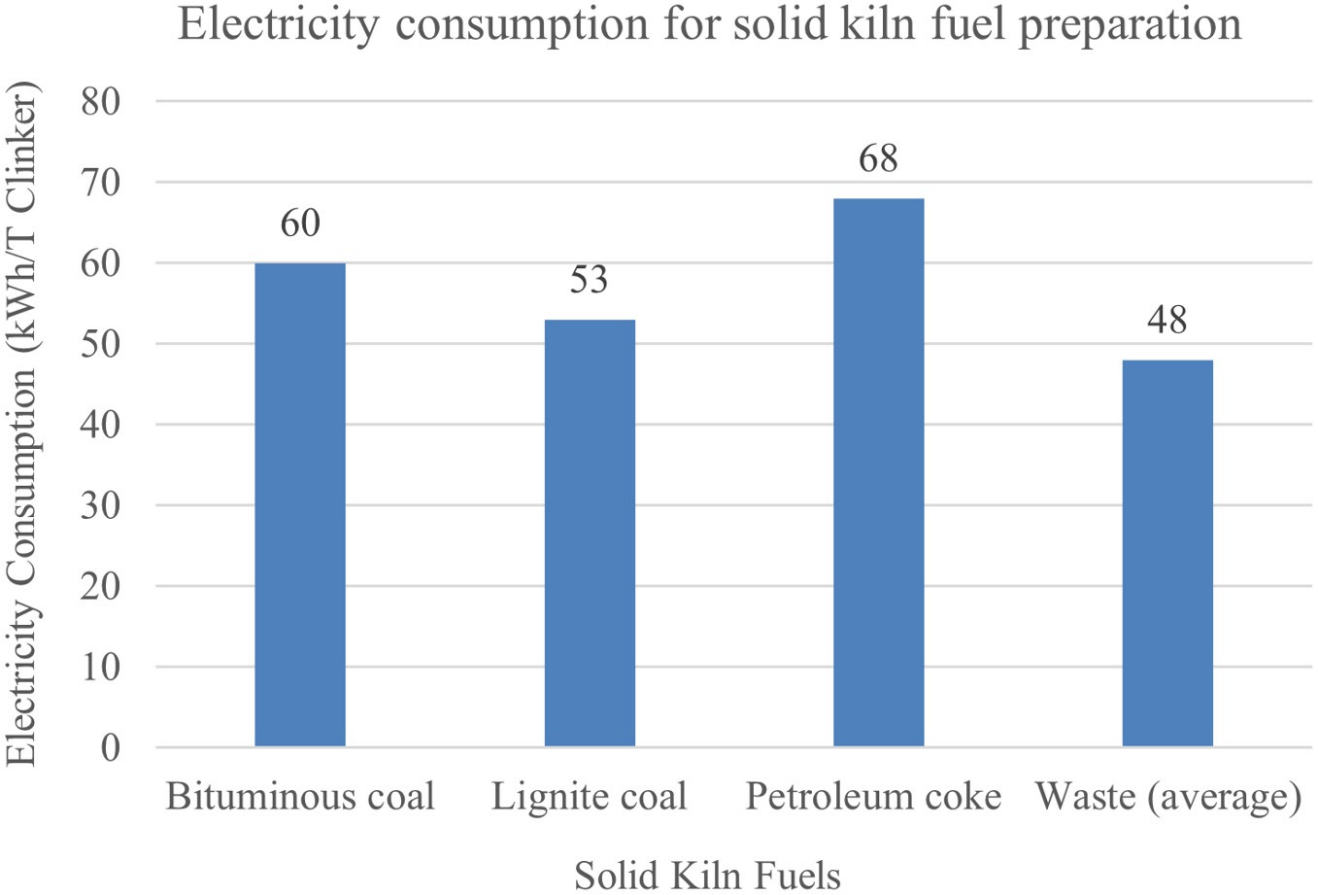
Electricity consumption for preparation of solid fuels [50, 55, 56].

**Fig 3:**
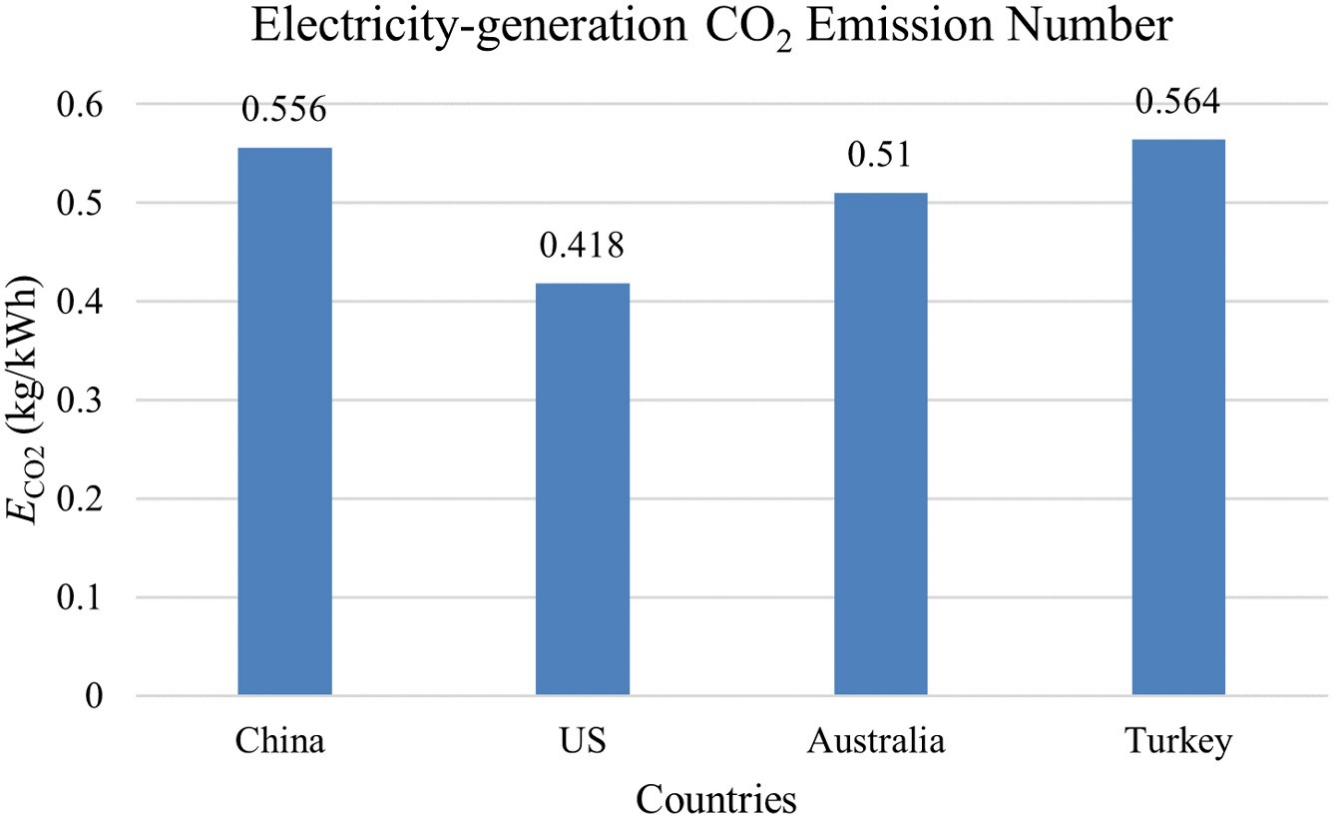
Calculated $E_{{CO}_{2}}$ for the four countries.

The electricity CO_2_ emission number (E_Elec_) is caused by the electricity consumption in pyroprocessing, which can be separated into two parts: the electricity used for operating the kiln, and for milling and drying the solid fuels (solid fuel preparation), such as coal and coke. Solid fuels are frequently used in the cement industry, but solid fuels must be milled and dried prior to use in the kiln, and this translates into additional consumption of electricity, as shown in Fig 2. Also, Table 4 gives the kiln operation electricity requirement. The total consumed electricity is generated in the electricity plants by consuming different types of fuels, emitting CO_2_. Then, Table 5 gives the electricity generation by energy sources for the four countries and the fuel-CO_2_ intensity of electricity generation by resources, and Fig 3 gives the calculated power-generation CO_2_ emission factor based on data given in Table 5. According to Eq (4), *Electricity*_*K*_ can be found in Table 4; *Fuel*_*%*_ can be seen in Table 1; *ECFKFP*_*i*_ is given in Fig 2; and $E_{{CO}_{2}}$ is calculated and listed in Fig 3, which is nearly in consistent with the literature [53, 54]. Thus, according to Eq (4), the calculated ECENs are 69, 35, 32, and 49 kgCO_2_/t clinker for China, the US, Australia, and Turkey, respectively.

**Table 4:** Electricity consumption by the kilns [5, 33, 50].

	Electricity Use (kWh/t clinker)		
Technology Options	Avg	Max	Min
**Wet kiln**	21	25	5
**Shaft kiln** ^a^	22	\	\
**Long dry kiln**	25	30	20
**Preheater kiln**	25	25	25
**Preheater/precalciner kiln**	25	25	25
**US Average kiln**	25	25	25
**China Average kiln** ^a^	25	\	\

^*a*:^Estimited by the authors based on data collected from [5, 14, 16].

**Table 5:** Power generation by source (%) for the four countries and the fuel-CO_2_ intensity (kg/kWh) [5, 32, 57, 58].

Country	Oil	Natural Gas	Bituminous Coal	Lignite Coal	Raw Coal	Nuclear Power	Hydropower	Renewable Energy	Others
**China**	0.1	3.1	\	\	64.7	4.8	17.8	9.5	\
**United States**	0.5	38.6	24.0	\	\	19.3	6.2	11.1	0.3
**Australia**	\	37.35	34.75	\	\	\	17.53	8.64	0.68
**Turkey**	3	49	\	28	\	\	19	1	\
**CO**_**2**_ **(kg/kWh)**	0.80	0.48	0.95	1.09	0.84	\	1.12E-03	6.6E-03	\

### 3.4 PCEN calculation

**Fig 4:**
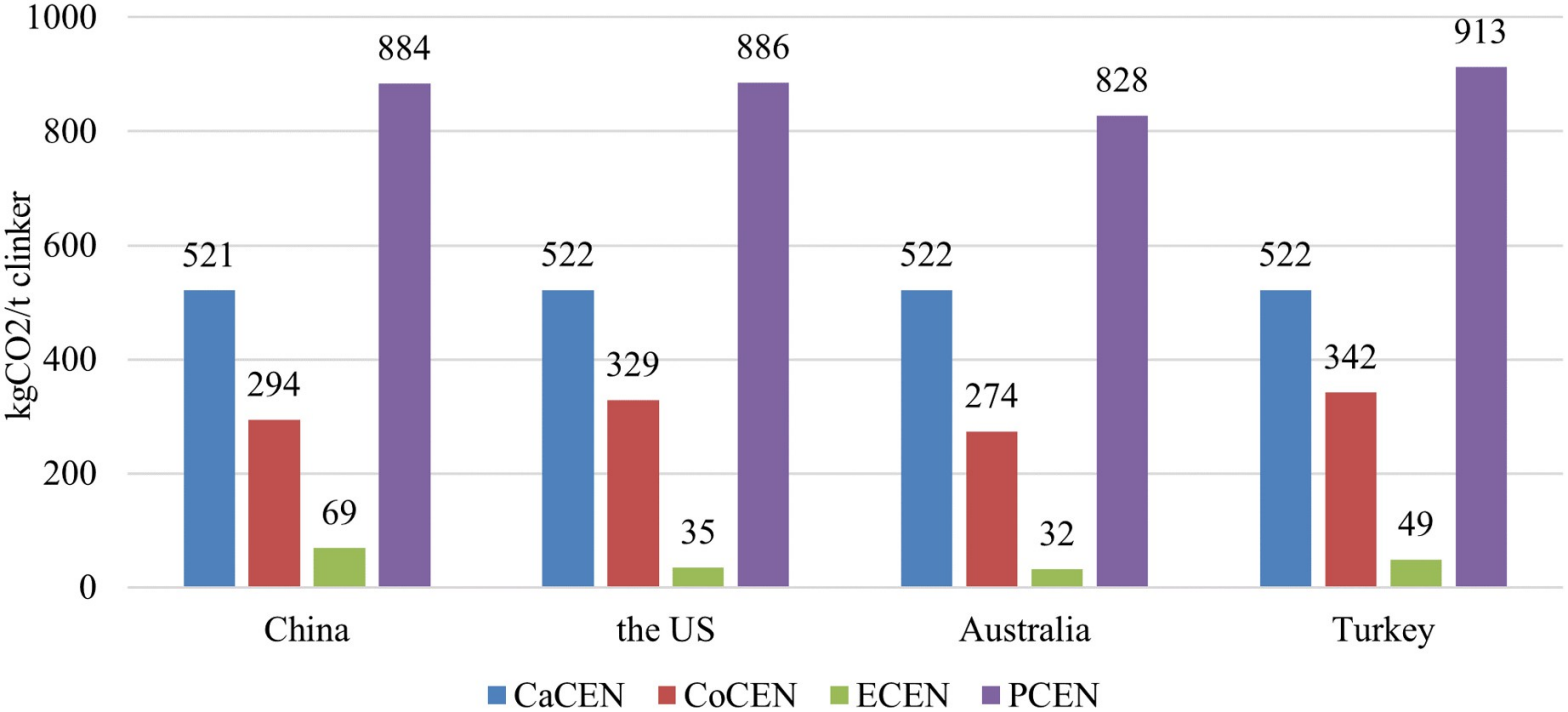
Calculation results of the four countries.

After estimating the CaCENs, CoCENs, and ECENs of the four countries, the PCENs (E_P_) can be calculated using Eq (1). The calculated PCENs are 884, 886, 828, and 913 kgCO_2_/t clinker for China, the US, Australia, and Turkey, respectively, as shown in Table 6 and Fig 4. Taking China as an example, the CaCEN, CoCEN, and ECEN represent 58.94%, 33.26%, and 7.81% of the PCEN, respectively, which is nearly in consistent with the research results of Cao et al. [59] and Supriya et al. [60], and hence the accuracy of the research result in this study can be verified.

**Table 6:** PCENs (*E*_*P*_) of the four countries (kgCO_2_/t clinker).

Country	China	the US	Australia	Turkey
**CaCEN (*E***_***Cal***_ **)**	521	522	522	522
**CoCEN (*E***_***Com***_ **)**	294	329	274	342
**ECEN (*E***_***Elec***_ **)**	69	35	32	49
**PCEN (*E***_***P***_ **)**	884	886	828	913

Note: CaCEN is calcination CO_2_ emission number; CoCEN is combustion CO_2_ emission number; ECEN is electricity CO_2_ emission number; PCEN is pyroprocessing CO_2_ emission number.

### 3.5 Sensitivity analysis for the PCEN calculation

Sensitivity analysis refers to the analysis method that systematically changes the uncertainty factors in the model to assess the impact of these changes on the model output [61, 62]. This analysis method can help researchers or decision-makers to deeply understand the robustness and reliability of the model, reveal potential risk factors, and improve the reliability and predictability of decision-making. For this study, the most significant uncertainty in PCEN calculation arises from the input data, and hence it is necessary to discuss the impact of the input data sensitivity on PCEN calculations, which mainly comes from three aspects, the CaCENs, CoCENs, and ECENs introduced in Sections 3.1–3.4.

Regarding the input data for CaCEN calculations, although CaCENs represent a significant portion of PCENs, the variation among CaCENs is not significant, as discussed in Section 3.1. Taking CaCEN for China as an example, the CaCENs can be different in some published articles. For instance, Liu et al. [35] reported a minimum value of 495 kgCO_2_/t clinker, while Lei et al. [39] proposed a maximum value of 550 kgCO_2_/t clinker. Then, the impact of CaCENs on PCEN can be identified, and the calculation results are presented in Table 7. It is found that the sensitivity of CaCENs on PCENs is relatively small, ranging from -2.94% to +3.28%.

**Table 7:** Influence of the CaCEN on PCEN.

CaCENs (kgCO_2_/t clinker)	PCENs (kgCO_2_/t clinker)	Fluctuation ranges of PCENs
**495**	858	-2.94%
**521**	884	0.00%
**550**	913	+3.28%

For CoCEN calculations, thermal energy consumption (*Energy*_*T*_) represents the primary source of uncertainty, as the energy structure tends to remain relatively stable in the short term. As discussed in Section 3.2, in the case of CoCEN of the US, the maximum thermal energy consumption is 3.8, while the minimum one is 3.1. Based on these maximum and minimum values, the sensitivity of CoCEN on PCEN is calculated and presented in Table 8, showing a range of -4.18% to +3.16%.

**Table 8:** Influence of the CoCEN on PCEN.

*Energy*_*T*_	CoCENs (kgCO_2_/t clinker)	PCENs (kgCO_2_/t clinker)	Fluctuation ranges of PCENs
**3.1**	292	849	-4.18%
**3.5**	329	886	0.00%
**3.8**	357	914	+3.16%

For ECEN calculations, given that the energy structure tends to remain relatively stable in the short term, the characteristics of kilns may be the major factor impacting PCENs. It is known that China’s economic and technological development is uneven, with significant regional differences. It can be assumed that some developing regions in China are still using long dry kilns with high electricity consumption (20–30 kWh/t clinker), while some developed areas have upgraded their cement industry, resulting in a higher prevalence of wet kilns and lower electricity consumption (5–25 kWh/t clinker). Thus, the electricity used by the kiln (*Electricity*_*K*_) may be ranged form 5 to 30 kWh/t clinker. Thus, according to the sensitivity of ECEN on PCEN shown in Table 9, the impact is in a range of -1.24% to +0.45%.

**Table 9:** Influence of the ECEN on PCEN.

*Electricity*_*K*_	ECENs (kgCO_2_/t clinker)	PCENs (kgCO_2_/t clinker)	Fluctuation ranges of PCENs
**5**	58	873	-1.24%
**25**	69	884	0.00%
**30**	73	888	+0.45%

To conclude, the fluctuation of PCENs impacted by the input data is in a range of -8.36% to +6.89% by the summary of the three aspects (CaCENs, CoCENs, and ECENs), which shows a minor sensitivity and will not influence the research results of this study significantly.

## 4. Discussion of impacted factors and common CE reduction measures

This section discusses the factors influencing CO_2_ emissions during pyroprocessing and accordingly gives common measures that can be used by all the countries to reduce pyroprocessing CO_2_ emissions.

### 4.1 Calcination CEs

The CaCEN (E_Cal_) is relatively stable as it is a series of chemical reactions, and the carbon element obeys the element balance before and after the chemical reactions [5, 35]. However, there are still factors that can influence the CaCEN. The first factor is the substitution of raw materials, such as limestone, sandstone, and clay, put into the kilns, which can significantly influence the factors of *F*_*CaO*_ and *R*_*D*_, and then influences the calculation results of CaCENs, as introduced in Section 3.1. For instance, steel slag and coal fly ash can be used to replace limestone and clay to reduce CO_2_ emissions, respectively. The current average steel slag and coal fly ash replacement levels in China is currently 1.8% and 1.2%, respectively, and the average substitution rate of the whole raw materials is about 1.3%, which can reduce the CaCEN by 3.6kgCO_2_/t clinker [14]. If the supply of the substitution materials is sufficient, the replacement level can be enhanced to 18% for the best practice level, and the CaCEN can be reduced significantly. The second factor that can slightly influence CaCEN could be the kiln type, but this influence is nearly neglected. Gao et al. [14] proved that the fluctuation ranges of CaCEN for preheater kilns, shaft kilns, and other kilns are all less than 3% in China from 1980 to 2014, and this kiln’s influence can be further ignored as the preheater kiln currently has a production of more than 92% in all of the four countries [5, 14, 16, 32], which means the kiln improving space is quite narrow. Also, Gursel [5] showed that the CaCENs for the wet kiln, the long dry kiln, the preheater kiln, and the preheater/precalciner kiln are almost the same in the US and Turkey. Thus, enhancing the level of the substitution of raw materials with non-carbonate or low-carbonate materials is a considerable and effective measure for reducing the CaCEN for all countries, while the measure of kiln improvement is not appropriate unless there is a boom of kiln technology in the short future.

### 4.2 Fuel combustion CEs

The factors that affect the CoCEN are the fuel type, the proportion of each selected fuel, and the kiln type. For the fuel type, it can be seen from Table 2 that the CO_2_ emission per HHV of different fuels can be quite different. For example, the CO_2_ emission per HHV of lignite coal is the highest (196kg/MJ), which is nearly four times that of natural gas (51kg/MJ), and hence if natural gas is selected as the fuel instead of lignite coal, CoCEN can be significantly reduced. The proportion of each selected fuel also influences the fuel combustion CEs. For example, it can be easily understood that enhancing the proportions of natural gas or reducing the proportion of lignite coal will result in a lower CoCEN. Last but not least, Table 3 shows that the advanced preheater kilns require only around 56% of the thermal energy compared with the outdated wet kilns, leading to much lower CO_2_ emissions, and hence the kiln type is the third factor that can influence the CoCEN. However, the kiln improvement for some countries can be quite limited under the currently available technology. For example, it can be seen in Table 3 that the average thermal consumption by kilns in the US (3.5 MJ/kg clinker) is as same as the one by preheater kilns (3.5 MJ/kg clinker), which means that the replacement of outdated kilns with advanced preheater kilns has nearly been completed in the US, whose circumstance is as same as that of China. The National Bureau of Statistics of China [17] reported that the preheater kilns produced 92% of China’s clinker in 2012 (increased from 40% in 2005). Thus, reducing combustion CO_2_ emissions by increasing the proportion of advanced kilns has been quite limited in countries like the US and China unless there is a boom in kiln technology in the short future. Therefore, using lower CO_2_-intensity fuels (such as natural gas) instead of high CO_2_-intensity fuels (such as lignite coal) is a reasonable and effective measure for reducing the CoCEN for all countries.

### 4.3 Electricity CEs

Since preheater kilns have dominated clinker production, and the electricity consumption by different types of kilns is almost the same according to Table 4, the kiln type is not a considerable factor that can influence the electricity CO_2_ emission number. On the contrary, the ECEN can be influenced by the fuel type and the country’s electricity grid structure. According to Fig 2, different solid fuels result in different quantities of electricity consumption for solid kiln fuel preparation. Moreover, if natural gas is used to replace solid fuels in pyroprocessing, the electricity consumption for solid kiln fuel preparation can be removed, leading to the reduction of electricity CO_2_ emissions. Furthermore, as shown in Table 5, it can be found that the CO_2_ emissions from electricity generation by nuclear power, hydropower, and renewable energy are almost negligible, and even for thermal power generation, the CO_2_ emission intensity of natural gas is almost half as much as coal. The CEFs of clean fuels are relatively negligible compared to the thermal ones. Thus, the different electricity grid structures result in quite different ECENs in the four countries (from 32 to 69 kgCO_2_/t clinker). Therefore, reducing the proportions of solid fuels used in pyroprocessing and improving the electricity grid structures are two effective strategies for all countries to reduce ECENs.

### 4.4 Reduction measures with a global context

From a global perspective, in addition to the reduction measures proposed in this section, more common CE reduction measures using innovative technologies are introduced, which can be applied to countries that are categorized into four aspects based on their economic growth, industrialization, and urbanization [63]. These reduction measures are thermal efficiency improvement (TEI), energy structure improvement (ESI), carbon capture and storage (CCS), and supplementary cementitious materials (SCMs) [52, 63, 64], which can be used for most countries as common reduction measures, while the effect of these reduction measures can be different for countries with different characteristics. The four aspects of countries are developed countries, China, rapid cement developing countries such as India and Vietnam, and potential cement developing countries.

For developed countries, characterized by strong economic performance, high cement demand was experienced before the 1990s, followed by a significant decrease in demand [5]. In these countries, measures like TEI, ESI, CCS, and SCMs can be implemented to reduce carbon emissions from the cement industry based on the specific characteristics of each country. For example, Tanaka and Managi [65] indicated that energy efficiency in the Japanese cement industry can be improved through industrial agglomeration, thereby reducing CEs from the sector. CCS technology is also applied in many EU countries, such as Italy, Spain, Sweden, and so on, for cement CE reductions [51]. Developing countries such as China, which not only has the largest cement production globally but also exhibits rapid economic, industrialization, and urbanization growth, achieved peak cement production in 2014 and has since experienced a decline [63], and hence is classified as the 2^nd^ country aspect. As China is one of the sample countries, the discussion will be presented in Section 5 instead of presented here repeatedly. Examples of the third aspect of countries are India and Vietnam. Due to limitations in investment in cleaner cement production, measures such as ESI may not effectively reduce cement carbon emissions in the short term, while CCS and SCMs may represent more viable pathways for reductions in these countries. For example, Lau and Tsai [66] investigated the role of carbon capture and storage (CCS) in decarbonizing Vietnam’s cement industry, aiming to enhance the cleanliness and sustainability of cement production. Moreover, Karadumpa and Pancharathi [67] conducted a study to compare the carbon emissions of blended cement with those of ordinary portland cement (OPC) in India, finding that blended cement has significant potential for reducing CEs through the use of SCMs. The last type of countries consists of most countries in the Global South, such as Indonesia, Myanmar, Egypt, and Tanzania, where the development of the cement industry lags behind that of the aforementioned countries but holds significant potential for future growth [63]. In these countries, SCMs may be a more viable way to reduce CEs for the cement industry. For instance, Salem et al. [68] utilized Egyptian cornstalk ash as a replacement for cement to achieve more sustainable concrete. Thus, the application of common reduction measures such as TEI, ESI, CCS, and SCMs can effectively mitigate CEs in the cement industry at a global level, and each country can choose appropriate measures based on its own characteristics.

## 5. Discussion of custom CE reduction measures

An effective CE reduction measure should adapt to the country’s own characteristics, such as natural resources, economic conditions, policies, and so on. A measure that is suitable for one country may not be appropriate for another one. This section analyses the advantages and disadvantages of each country for PCE reduction and discusses the PCE reduction potential for the sample countries. Then, further custom measures are provided to each of the four selected sample countries based on their characteristics.

### 5.1 PCE reduction measures for the US, Australia, and Turkey

It is well known that the US is a country rich in resources, especially in petroleum-related resources and natural gas, and is exporting large quantities of these resources every year. However, the major combusted fuel in kilns used in the US is still bituminous coal which has a relatively high CEF according to Table 1. Oppositely, Australia is performing well in using natural gas to release 34% of the thermal energy used in kilns, which leads to the combustion CO_2_ emission number being 19% lower than that of the US. On the contrary, according to Table 6, the US’s ECEN is relatively low (35kgCO_2_/t clinker) because of the relatively high proportion of nuclear power, but there is still room for improvement. It is reasonable to replace bituminous coal with natural gas in the kiln fuel mix to eliminate the electricity consumption for solid kiln fuel preparation to further reduce ECEN. Thus enhancing the proportion of natural gas in the kiln fuel mix will be a reasonable and effective strategy for the US to reduce combustion and electricity CO_2_ emissions depending on sufficient natural gas reserves. Furthermore, if natural gas is used instead of bituminous coal for power generation to improve the electricity grid, the US’s ECEN can be further reduced.

Australia also has performed well in electricity CO_2_ emission reduction with only a value of 32 kgCO_2_/t clinker because of the high proportion of natural gas used in the kiln fuel mix and power generation. However, under the restriction of nuclear power, increasing the proportion of natural gas used in kiln combustion and power generation could be limited because of the cost, resulting in the improvement in further reducing CoCEN and ECEN could be limited. On the contrary, if a considerable proportion of bituminous coal can be replaced by natural gas, the US’s PCEN even can be lower than that of Australia because of the use of nuclear power.

For Turkey, most of the kiln fuel mix is constituted of lignite coal and petroleum coke, whose CEFs are quite high. Thus, Turkey has the highest CoCEN (342 kgCO_2_/t clinker), which is 24% higher than that of the lowest one (Australia) in the four countries. Moreover, Turkey’s electricity consumption for the solid kiln fuel preparation is also the highest because no natural gas is used in the kiln according to Table 1. In addition, although there is a high proportion of natural gas used in power generation, a large amount of lignite coal which has the highest CEF is also combusted for power generation, resulting in a relatively high ECEN. The improper fuel selections resulted in the highest PCEN in the four sample countries. Thus, increasing the proportion of natural gas in the kiln fuel mix and reducing the use of lignite coal in power generation are two effective and urgent measures to reduce the PCEN for Turkey.

### 5.2 PCE reduction measures for China

As China has more than half of the world’s cement production and is facing the largest CO_2_-reduction challenge because of the largest CO_2_ emissions, it is quite urgent to propose effective and efficient CE reduction measures for China. China’s challenge is not only because of the huge industrial volume but also because of the inappropriate energy structure. S1 and S2 Tables (in the appendix) list China’s total primary energy production and total energy consumption, respectively. It can be seen from S1 and S2 Tables that the total primary energy production and consumption are 3.57E+09 and 4.48E+09 tce, respectively, which means there is a huge amount of 9.1E+08 tce energy gap. Also, according to S1 and S2 Tables, coal production in China can nearly satisfy the energy consumption (still with an energy gap of 9E+07 tce), but the consumptions of petroleum and natural gas are around 3 and 2.5 times as much as the production of these two sources with energy gaps of 6.6E+08 and 1.6E+08 tce, respectively. As coal is the most important fossil fuel resource in China, it has undertaken more than 63% of energy consumption in the past 30 years. On the contrary, renewable energy and natural gas are less than a share of 5% and 9%, respectively, and the other 21% is supplied by oil. The huge energy gap is not only because of the requirement but also because China lacks the resources of oil and natural gas, an inherent inappropriate energy structure. The inappropriate energy structure is even much more serious in the cement industry, as nearly total energy consumption in pyroprocessing is afforded by coal. As a result, the PCEN is high, especially in North China, and the annual financial cost of pollution is about 400 billion dollars, which accounts for around 10% of China’s GDP annually [58]. Thus, as the largest manufacturing power, China is currently facing the largest environmental challenge that humans have ever met.

**Fig 5:**
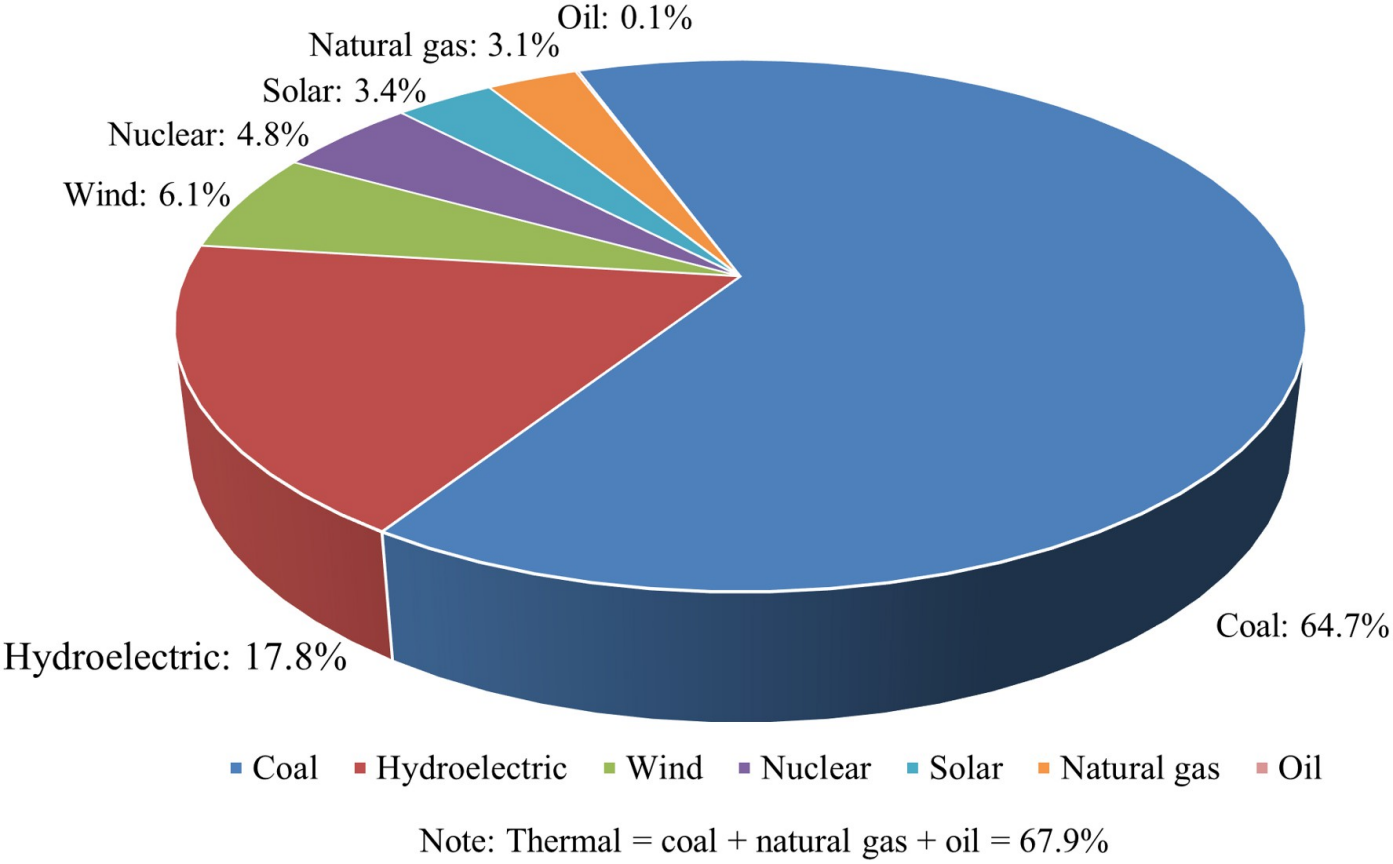
China’s power generation by sources in 2020.

According to Fig 5, 64.7% of China’s power generation depends on raw coal which has a high CEF; hence China has the highest ECEN among the four sample countries. However, increasing the proportion of natural gas in the fuel mix in pyroprocessing or power generation like the other three sample countries is not an appropriate measure for China, and the reasons are explained below. S3 Table (in the appendix) gives the imports of major energy products of China, and it can be seen that the top three major imports of energy products are coal, crude oil, and natural gas. For petroleum-related products, the import quantity is still much less than the energy gap, which means that a large part of the imported coal (or equivalent coal products) is transferred to petroleum-related products, as the import quantity of coal is larger than the energy gap. For natural gas, the import quantity is also huge because of the large energy gap in natural gas. Therefore, from China’s energy structure shown in S1–S3 Tables, the reason for using coal as the major fuel for pyroprocessing combustion and power generation in China is found, the inherent inappropriate energy structure. Thus, considering the huge pressure under imports of petroleum-related products and natural gas, substituting coal for natural gas to reduce the PCEs is not quite realistic in the short term. Additionally, increasing the import of natural gas is also not realistic, because this strategy will lead to China’s higher financial pressure, and also cannot be achieved in the short term because China’s energy import structure has been stable in the past 10 years (2010–2019) according to S3 Table. Furthermore, the current import of fossil energy has been affected by uncertainties, and the expenditure is even uncertain. For instance, the import could be reduced or even stopped because of certain circumstances, such as the unpredicted huge disease disaster that lasted for 3 years and the unexpected war in Eastern Europe. Moreover, the price of oil could wave between 30 and 147 dollars during the 2008 financial crisis, which was unpredictable and could cause economic problems [69]. Thus, increasing the proportion of natural gas usage is not a reasonable measure for China because of the insufficient natural gas reserves.

To reduce CO_2_ emissions during pyroprocessing in China’s cement industry, the optimal strategy is to improve China’s energy structure, especially the energy structure for power generation. S4 and S5 Tables (in the appendix) state the cement production in each region of China mainland and the regional power generation (sorted by the thermal power production), respectively. The authors find that the top 10 cement production regions and thermal power generation regions are highly coincidental, such as Jiangsu, Shandong, Guangdong, Zhejiang, Anhui, Henan, etc., most of which are in East China and also the top 10 GDP regions of China. This means that most of the electricity consumption during pyroprocessing in the high cement-production regions is provided by thermal power, resulting in a large ECEN which is almost twice as much as the ones of the US and Australia. After finding the reasons, the obvious method to reduce the ECEN is to improve the electricity grid by increasing the proportion of renewable energy, especially in the top 10 cement production regions. For instance, the average CEF of the national power grid in 2022 is 0.57 tCO_2_/MWh [57, 69], but for Shandong province, this factor is 0.725 tCO_2_/MWh calculated by the authors based on the data recorded in the literature of [5, 58], which is much higher than the national value because of the 88.5% thermal power [58]. However, the CEF of Sichuan province is calculated as only 0.12 tCO_2_/MWh, as 85% of the power is hydroelectric [58], whose CEF can be nearly ignored compared with thermal power, resulting in the electric CEF in Sichuan is around only 1/6 of that in Shandong Province. Thus, using hydroelectric power is an effective measure for some regions of China to reduce CO_2_ emissions. However, hydroelectric power also has its limitations. It can be found in S4 Table (in the appendix) that hydro-power has been a large proportion in some regions such as Sichuan, Yunnan, and Hubei, but hydro-power heavily depends on the geographical conditions and cannot be achieved in most of the top 10 cement production and thermal power generation regions (simplified as the Regions). This circumstance is also similar to wind power which is only abundant in regions that have relatively low cement production, such as Inner Mongolia, Xinjiang, and Gansu. Thus, hydroelectric and wind power are effective CE reduction measures for some geographically appropriate regions in China, but they cannot solve the essential issues of the Regions.

Because of the limitations of hydroelectric and wind power, one option is to study the US to use more nuclear power to reduce the CEs. However, in consideration of the sensitivity of nuclear power, developing solar power can be a more reasonable method for the Regions to improve the energy structure to reduce CEs and get a sustainable energy resource (China’s coal and oil resources are predicted to dry up by 2050 [58, 70]). Fortunately, China has the largest photovoltaic (PV) production in the world, and the grid-connect photovoltaic generation (GPG) technique has been currently used for solar power generation. The housetop and south wall areas, most of which are in East China, are more than 9 billion m^2^ in China now, and if these areas are used to install photovoltaics and generate solar power, the capacity is predicted as 100 GWh [70], which represents around 7% of the country’s total electricity generation and can significantly reduce ECEN from thermal power. Moreover, a more advanced wind-solar hybrid system that can overcome the drawback that solar power depends on climatic conditions is being developed rapidly. As China has abundant wind resources in the seaboard areas, such as Jiangsu, Zhejiang, Shandong, and Guangdong, this hybrid system can be used to further reduce the proportion of thermal power in these large cement production regions. In addition, solar energy hearth and solar heating buildings are also being used, which can save plenty of coals and natural gas used in people’s daily lives, and then the saved natural gas can be transferred to the cement industry to further reduce CoCEN. Furthermore, new measures learned from the techniques of solar water heaters and solar heating buildings can be developed to use solar energy directly in pyroprocessing to replace fossil energy in the near future to significantly reduce CoCEN. Thus, vigorously increasing the proportion of solar energy usage can effectively and efficiently reduce combustion and electricity CO_2_ emissions in pyroprocessing for China’s cement industry.

For calcination CO_2_ reduction, the most effective method is to continually enhance the level of the use of alternative raw materials with non-carbonate or low-carbonate materials as introduced in Section 4.1, and improving kiln techniques is another option. Kyoto Protocol [71] listed measures to reduce calcination CO_2_ emissions, and then Gao et al. [14] evaluated these measures and stated that seal replacement in rotary kilns, optimizing heat recovery in kilns, and kiln shell heat loss reduction are the best three CO_2_ abatement measures with the lowest net costs. In summary, the core idea for reducing pyroprocessing-related or even whole industrial CO_2_ emissions is to improve China’s energy structure by using renewable energy, especially solar energy. It also can be predicted that the CEs can be greatly reduced if the measures or strategies introduced in this study can be applied in China in the near future, which also can be used for reference by other countries.

### 5.3 CE potential of sample countries

In summary, the PCENs of the four countries are not quite different according to Table 6, but all four countries have the potential to reduce CO_2_ emissions in pyroprocessing or even the whole industry. To be more specific, the US has the best CO_2_ reduction potential among the four countries. At first, nearly no natural gas is currently combusted in the kilns in the US which has abundant natural gas reserves, so the US’s CoCEN can be reduced dramatically if a relatively large proportion of natural gas is used in the fuel mix. In addition, the US’s ECEN is the lowest among the four countries, because nearly 20% of the power is generated by nuclear power, which cannot be achieved by Australia and Turkey under the restrictions of the policies, and also hardly can be reached by China in a short term. This ECEN can be further reduced if more natural gas is used for power generation in the US. Thus, the US has the best CE potential because of its appropriate inherent energy structure. Similarly, Australia also has a good inherent energy structure, but its CE potential possibly is limited because a relatively high proportion of natural gas has been used in pyroprocessing and power generation, which means further enhancing the proportion of natural gas could be uneconomical. However, Australia has been performing well in controlling PCEN and thus does not have much pressure on CE reductions in the short term. On the contrary, although Turkey has the highest proportion of natural gas in power generation, the country still has the highest PCEN among the four countries because a large amount of lignite coal is used. If lignite coal is replaced by other types of clean energy sources, such as natural gas or renewable energy, the PCEN can be significantly reduced. Among the four countries, China has the most challenging circumstance in reducing PCEs, not only because of the largest cement production but also because of the CO_2_ reduction requirement of the country’s two-carbon goals. China has to consider improving the energy structures both in the cement industry and power generation by using renewable energy sources instead of coal to reduce PCEN.

### 5.4 Research boundaries of this study

The boundaries of this study are stated in this section. At first, this study focuses on the CEs in the pyroprocessing procedure which accounts for around 90% of the total CEs during all cement production procedures, while other procedures, such as quarry, transportation, milling, grinding, blending, and so on, are not included in this study. This is because the CE reduction measures in pyroprocessing are the most effective and efficient. Further investigation into CE reduction measures for other procedures can be also considered (although the CO_2_ emissions in these procedures account for only around 10%) to complete the CO_2_ reduction chain for the whole cement production in the future. Secondly, a small amount of data cannot be collected from multiple sources because of the limitations, which can be improved by extending the existing database. Furthermore, it cannot be denied that the energy structure of a country greatly influences the change in PCEN within that country, and each country has differences in its energy systems and cement production situations. More sample countries are required to be investigated to provide corresponding custom strategies for further global CE reduction in the cement industry. Custom measures based on the characteristics of national energy systems or other systems can be applied to reduce CEs in the cement industry. The model proposed in this study cannot only be used in the four sample countries but can be propagated to other countries because the cement production procedures are generally similar. This means that the proposed model can be used to calculate CEs in the pyroprocessing procedure for most countries if accurate input data is available, and hence the boundary of this proposed model is for most countries instead of limited sample countries which are selected as they are representative because of their characteristics. The model and measures proposed in this study will help with the CE reduction in the world’s cement industry, and the collected data and calculated results can be used to further cement CE reduction research in the future.

## 6. Conclusion and prospects

The carbon emission from the pyroprocessing procedure of cement production accounts for around 5% of global CEs and has been an obstacle to the world’s environmental protection, and hence looking for measures to effectively reduce PCEs for the world’s cement industry becomes quite urgent and necessary. This study uses an analytical model to calculate the PCENs of countries to identify the impacted factors and propose tailored measures of PCE reduction for countries based on their own characteristics. More specifically, PCENs of four sample countries, the US, China, Australia, and Turkey are calculated using the analytical model based on the collected data, and the results are comprehensively analyzed and discussed to find the CE impact factors. Then, the common and custom CE reduction measures are correspondingly proposed for the sample countries based on the countries’ characteristics. Existing data is collected in this study to ensure the integrity of the system boundary and guarantee the model can be applied accurately to obtain reliable results.

The results show that the CaCENs of the four sample countries are almost the same because this chemical reaction is stable and follows the principle of element balance. The CoCENs are 294, 329, 274, and 342 kgCO_2_/t clinker for China, the US, Australia, and Turkey, respectively. Australia has the lowest CoCEN because of the high proportion of natural gas consumption. The calculated ECENs are 69, 35, 32, and 49 kgCO_2_/t clinker for China, the US, Australia, and Turkey, respectively. China has the highest ECEN because of the highest proportion of thermal power. As a result, the PCENS are calculated as 884, 886 828, and 913 kgCO_2_/t clinker for China, the US, Australia, and Turkey, respectively. It is found that Australia is performing best with the lowest PCEN, while Turkey has the largest one. Factors influencing PCENs are subsequently analyzed and discussed, and the corresponding CE reduction measures are proposed. The authors find that enhancing the level of the substitution of raw materials with non-carbonate or low-carbonate materials, using low CO_2_ intensity fuels for combustion, reducing the proportions of solid fuels used in pyroprocessing, and improving the electricity grid structures are effective measures for all the countries to reduce PCENs. For the four sample countries, the advantages and disadvantages of each country in PCEN reduction are discussed. The results show that the US has the largest potential for PCEN reduction because of its abundant energy resources and the use of nuclear power. Turkey also can dramatically reduce PCEN in the short term if the proportion of lignite coal can be largely reduced. On the contrary, China is facing the largest challenge in PCEN reduction because of the improper energy structure and large cement production. Thus, more attention is paid to China in this study for the purpose of giving reasonable measures to reduce PCEN in this country. It is found that China’s predicament is because of insufficient natural energy resources. More specifically, nearly 70% and 30% of the oil and natural gas have to be imported from overseas because of the local primary energy production gap, which results in the majority of energy consumption being from coal combustion. To overcome this large difficulty, the optimum strategy for China is to improve its energy structure by dramatically increasing the proportion of renewable energy, such as solar and wind power, and the details of corresponding measures for achieving this objective are also proposed. The collected data and analytical results given in this study can help engineers and researchers to further investigate techniques for CE reductions and provide policymakers the suggestions to further improve the energy conservation and emission reduction policies for cement-related industries in different countries. It can be predicted that the measures and strategies proposed in this work will facilitate the more effective and efficient reduction of CEs in real-world cement industrial practice for different regions and countries based on their own characteristics. Besides the four sample countries, the model proposed in this study can be applied to other countries if accurate data is available. Measures based on their own characteristics can also be applied to achieve CE reductions. Therefore, it is advisable to investigate the PCENs of more countries to further achieve sustainability in the global cement industry.

In the future, attention should also be paid to innovative technologies such as carbon capture and storage (CCS), alternative clinker materials, and other technologies related to the recycling of waste to further reduce CEs in cement industries. For example, Georgiades et al. [51] indicated that CCS aims to capture process-based CEs before or after fuel combustion, which can be used for the cement industry. Guo et al. [72] conducted a review of innovative CCS technologies at a global level to reduce CEs. Moreover, Antunes et al. [73] proposed a study on alternative clinker technologies for reducing carbon emissions in the cement industry and stated that the transition from clinker production to electrification may lead to the complete elimination of carbon emissions in heating clinker kilns. Tan et al. [74] assessed the potential for carbon emission reduction and found that alternative clinker materials are related to process-related emissions, which could achieve 30–39% reduction in cumulative emissions. Additionally, the recycling of waste has consistently been a prominent topic of interest within academic research. Zia et al. [75] used recycled steel fibers from waste tires instead of industrial steel fibers to enhance the strength performance of cement mortar. They found that recycled steel fibers from waste tires could increase the compressive strength, split tensile strength, and flexural strength of cement mortar by 46%, 50.6%, and 69%, respectively. Umar et al. [76] developed a sustainable concrete using ceramic waste powder and found that replacing 30% of the cement with ceramic waste powder could achieve the maximum compressive strength. Umar et al. [77] also found that replacing 30% of the cement with ceramic waste powder can improve mechanical and durability properties compared to conventional concrete. Overall, the concept of low-carbon development in the cement industry of each country has its unique characteristics and shares some common challenges [72]. Appropriate innovative technologies can be implemented to reduce CEs in the cement industry based on specific conditions of each country. The global cement industry is predicted to be more sustainable with the development of technologies and application of more innovative CE reduction measures.

## Supporting information

S1 Table China’s total primary energy production and its composition. (DOCX)

S2 Table China’s total energy consumption and its composition. (DOCX)

S3 Table Imports of major energy products of China. (DOCX)

S4 Table Cement production in each province on China mainland in 2014. (DOCX)

S5 Table Regional power generation in 2020 (Unit: Hundred million kilowatt hour). (DOCX)

S1 Data (XLSX)
