# Supplementary material for: Reducing carbon emissions in the cement industry using effective measures based on countries’ characteristics
Source: PLoS One. 2024 Nov 21;19(11):e0311859. doi: 10.1371/journal.pone.0311859 (PMC11581325; doi:10.1371/journal.pone.0311859)
Supplement: S1 Table — (DOCX) [file pone.0311859.s001.docx]

**Supplementary Information**

1. China's total primary energy production and its composition

Table S1. China's total primary energy production and its composition [1].

| **Year** | **Calorific Value Calculation** | | | | | | |
| --- | --- | --- | --- | --- | --- | --- | --- |
|  | **Total**  **Primary Energy**  **Production**  **(10^4^ tce)** | **Proportion (%)** | | | | | |
|  |  | **Raw Coal** | **Crude Oil** | **Natural**  **Gas** | **Primary**  **Electricity and**  **Other Energy** | **Renewable** | |
|  |  |  |  |  |  | **Hydro**  **Power** | **Nuclear**  **Power** |
| **2010** | 294807 | 80.7 | 9.8 | 4.3 | 5.2 | 3.0 | 0.3 |
| **2011** | 323045 | 81.9 | 9.0 | 4.3 | 4.8 | 2.7 | 0.3 |
| **2012** | 330203 | 81.0 | 9.0 | 4.4 | 5.6 | 3.2 | 0.4 |
| **2013** | 336452 | 80.4 | 8.9 | 4.7 | 6.0 | 3.4 | 0.4 |
| **2014** | 336314 | 79.2 | 9.0 | 5.0 | 6.8 | 3.9 | 0.5 |
| **2015** | 334162 | 78.2 | 9.2 | 5.2 | 7.4 | 4.2 | 0.6 |
| **2016** | 315217 | 76.7 | 9.0 | 5.7 | 8.6 | 4.6 | 0.8 |
| **2017** | 325917 | 76.6 | 8.4 | 6.0 | 9.0 | 4.5 | 0.9 |
| **2018** | 342312 | 76.6 | 7.9 | 6.0 | 9.5 | 4.4 | 1.1 |
| **2019** | 357130 | 76.2 | 7.6 | 6.3 | 9.9 | 4.5 | 1.2 |

**References**

1. National Bureau of Statistics. China Energy Statistical Yearbook (2020 Edition). Beijing: China Statistics Press; 2020.
