# Supplementary material for: Reducing carbon emissions in the cement industry using effective measures based on countries’ characteristics
Source: PLoS One. 2024 Nov 21;19(11):e0311859. doi: 10.1371/journal.pone.0311859 (PMC11581325; doi:10.1371/journal.pone.0311859)
Supplement: S2 Table — (DOCX) [file pone.0311859.s002.docx]

**Supplementary Information**

2. China's total energy consumption and its composition

Table S2. China's total energy consumption and its composition [1].

| **Year** | **Calorific Value Calculation** | | | | | | |
| --- | --- | --- | --- | --- | --- | --- | --- |
|  | **Total**  **Energy**  **Consumption**  **(10^4^ tce)** | **Proportion (%)** | | | | | |
|  |  | **Coal** | **Petroleum** | **Natural**  **Gas** | **Primary**  **Electricity and**  **Other Energy** | **Renewable** | |
|  |  |  |  |  |  | **Hydro**  **Power** | **Nuclear**  **Power** |
| **2010** | 343601 | 72.7 | 18.3 | 4.2 | 4.8 | 2.6 | 0.3 |
| **2011** | 370163 | 73.4 | 17.6 | 4.8 | 4.2 | 2.3 | 0.3 |
| **2012** | 381515 | 72.2 | 17.9 | 5.1 | 4.8 | 2.8 | 0.3 |
| **2013** | 394794 | 71.3 | 18.0 | 5.6 | 5.1 | 2.9 | 0.3 |
| **2014** | 402649 | 70.0 | 18.4 | 6.0 | 5.6 | 3.3 | 0.4 |
| **2015** | 406312 | 68.1 | 19.7 | 6.2 | 6.0 | 3.4 | 0.5 |
| **2016** | 410984 | 66.8 | 20.1 | 6.6 | 6.5 | 3.5 | 0.6 |
| **2017** | 423108 | 65.3 | 20.4 | 7.4 | 6.9 | 3.5 | 0.7 |
| **2018** | 435649 | 63.9 | 20.4 | 8.3 | 7.4 | 3.5 | 0.8 |
| **2019** | 447597 | 62.8 | 20.7 | 8.7 | 7.8 | 3.6 | 1.0 |

**References**

1. National Bureau of Statistics. China Energy Statistical Yearbook (2020 Edition). Beijing: China Statistics Press; 2020.
