# Supplementary material for: Reducing carbon emissions in the cement industry using effective measures based on countries’ characteristics
Source: PLoS One. 2024 Nov 21;19(11):e0311859. doi: 10.1371/journal.pone.0311859 (PMC11581325; doi:10.1371/journal.pone.0311859)
Supplement: S3 Table — (DOCX) [file pone.0311859.s003.docx]

**Supplementary Information**

3. Imports of major energy products of China

Table S3. Imports of major energy products of China [1].

| **Item** | **2010** | **2011** | **2012** | **2013** | **2014** | **2015** | **2016** | **2017** | **2018** | **2019** |
| --- | --- | --- | --- | --- | --- | --- | --- | --- | --- | --- |
| **Coal (10^4^ tons)** | 18307 | 22236 | 28841 | 32702 | 29122 | 20406 | 25555 | 27092 | 28210 | 29977 |
| **Coke and Semi-coke (10^4^ tons)** | 11 | 12 | 8 | 3 | \ | \ | \ | 1 | 9 | 52 |
| **Crude Oil (10^4^ tons)** | 23768 | 25378 | 27103 | 28174 | 30837 | 33548 | 38101 | 41946 | 46189 | 50568 |
| **Gasoline (10^4^ tons)** | \ | 3 | \ | \ | 3 | 17 | 21 | 2 | 45 | 33 |
| **Kerosene (10^4^ tons)** | 487 | 618 | 621 | 669 | 414 | 348 | 352 | 376 | 413 | 367 |
| **Diesel Oil (10^4^ tons)** | 180 | 233 | 91 | 27 | 47 | 43 | 92 | 75 | 71 | 119 |
| **Fuel Oil (10^4^ tons)** | 2299 | 2684 | 2683 | 2347 | 1785 | 1540 | 1174 | 1357 | 1666 | 1486 |
| **Liquefied Petroleum Gas (10^4^ tons)** | 327 | 350 | 359 | 452 | 739 | 1244 | 1679 | 1922 | 1966 | 2109 |
| **Other Petroleum Products (10^4^ tons)** | 1731 | 1648 | 1548 | 1924 | 1677 | 2083 | 2067 | 2396 | 2592 | 2288 |
| **Natural Gas (10^8^ m3)** | 165 | 312 | 421 | 525 | 591 | 611 | 746 | 946 | 1246 | 1332 |
| **Electricity (10^8^ kWh)** | 56 | 66 | 69 | 75 | 68 | 62 | 62 | 64 | 57 | 49 |

**References**

1. National Bureau of Statistics. China Energy Statistical Yearbook (2020 Edition). Beijing: China Statistics Press; 2020.
