# Supplementary material for: Reducing carbon emissions in the cement industry using effective measures based on countries’ characteristics
Source: PLoS One. 2024 Nov 21;19(11):e0311859. doi: 10.1371/journal.pone.0311859 (PMC11581325; doi:10.1371/journal.pone.0311859)
Supplement: S4 Table — (DOCX) [file pone.0311859.s004.docx]

**Supplementary Information**

4. Cement production in each province on China mainland in 2014

Table S4. Cement production in each province on China mainland in 2014 [1].

| **Regions** | **Production**  **(Mt)** | **Proportion (%)** | **Production ranking** | **Year-on-year growth (%)** | **Growth ranking** |
| --- | --- | --- | --- | --- | --- |
| **Mainland** | 2476 | 100 | \ | 1.7 | \ |
| **Jiangsu** | 194 | 7.84 | 1 | 3.66 | 12 |
| **Henan** | 169 | 6.86 | 2 | 1.66 | 14 |
| **Shandong** | 164 | 6.63 | 3 | -0.77 | 19 |
| **Guangdong** | 147 | 5.95 | 4 | 12.77 | 3 |
| **Sichuan** | 145 | 5.89 | 5 | 4.87 | 10 |
| **Anhui** | 129 | 5.21 | 6 | 1.61 | 15 |
| **Zhejiang** | 123 | 4.99 | 7 | -0.5 | 18 |
| **Hunan** | 120 | 4.85 | 8 | 5.75 | 8 |
| **Hubei** | 117 | 4.71 | 9 | 3.12 | 13 |
| **Guangxi** | 106 | 4.3 | 10 | -0.04 | 17 |
| **Hebei** | 106 | 4.29 | 11 | -15.14 | 30 |
| **Jiangxi** | 98 | 3.96 | 12 | 6.32 | 7 |
| **Yunnan** | 95 | 3.83 | 13 | 4.06 | 11 |
| **Guizhou** | 94 | 3.79 | 14 | 15.48 | 2 |
| **Shaanxi** | 91 | 3.67 | 15 | 5.19 | 9 |
| **Fujian** | 77 | 3.12 | 16 | -1.37 | 21 |
| **Chongqing** | 67 | 2.69 | 17 | 9.44 | 5 |
| **Inner Mongolia** | 63 | 2.53 | 18 | -2.02 | 23 |
| **Liaoning** | 58 | 2.34 | 19 | -4.24 | 24 |
| **Gansu** | 49 | 1.99 | 20 | 9.91 | 4 |
| **Xinjiang** | 48 | 1.94 | 21 | -8.66 | 27 |
| **Jilin** | 47 | 1.88 | 22 | 1.54 | 16 |
| **Shanxi** | 45 | 1.83 | 23 | -7.71 | 26 |
| **Heilongjiang** | 37 | 1.48 | 24 | -9.15 | 29 |
| **Hainan** | 21 | 0.87 | 25 | 8.3 | 6 |
| **Qinghai** | 18 | 0.74 | 26 | -1.93 | 22 |
| **Ningxia** | 18 | 0.72 | 27 | -6.13 | 25 |
| **Tianjin** | 10 | 0.39 | 28 | -1.02 | 20 |
| **Beijing** | 7 | 0.28 | 29 | -18.84 | 31 |
| **Shanghai** | 7 | 0.28 | 30 | -8.71 | 28 |
| **Tibet** | 3 | 0.14 | 31 | 15.7 | 1 |

**References**

1. China Cement Association. China Cement Almanac 2014. Beijing: China Building Materials Industry Press; 2014.
