# Supplementary material for: Reducing carbon emissions in the cement industry using effective measures based on countries’ characteristics
Source: PLoS One. 2024 Nov 21;19(11):e0311859. doi: 10.1371/journal.pone.0311859 (PMC11581325; doi:10.1371/journal.pone.0311859)
Supplement: S5 Table — (DOCX) [file pone.0311859.s005.docx]

**Supplementary Information**

5. Regional power generation in 2020

Table S5. Regional power generation in 2020 (unit: hundred million kilowatt hour) [1].

| **Region** | **Total** | **Thermal** | **%** | **Hydro** | **%** | **Wind** | **%** | **Solar** | **%** | **Nuclear** | **%** |
| --- | --- | --- | --- | --- | --- | --- | --- | --- | --- | --- | --- |
| **Mainland** | 76264 | 51770 | 67.88 | 13553 | 17.75 | 4665 | 6.14 | 2611 | 3.42 | 3665 | 4.81 |
| **Shandong** | 5781 | 5116 | 88.50 | 9 | 0.16 | 259 | 4.48 | 206 | 3.56 | 191 | 3.30 |
| **Inner Mongolia** | 5703 | 4731 | 82.96 | 57 | 1.00 | 726 | 12.73 | 188 | 3.30 | 0 | 0 |
| **Jiangsu** | 5074 | 4290 | 84.55 | 32 | 0.63 | 229 | 4.51 | 167 | 3.29 | 356 | 7.02 |
| **Guang-dong** | 5048 | 3425 | 67.85 | 286 | 5.67 | 103 | 2.04 | 74 | 1.47 | 1160 | 22.98 |
| **Xinjiang** | 4052 | 3193 | 78.80 | 268 | 6.61 | 434 | 10.71 | 157 | 3.87 | 0 | 0 |
| **Shanxi** | 3395 | 2924 | 86.13 | 47 | 1.38 | 266 | 7.84 | 159 | 4.68 | 0 | 0 |
| **Anhui** | 2785 | 2531 | 90.88 | 66 | 2.37 | 57 | 2.05 | 130 | 4.67 | 0 | 0 |
| **Zhejiang** | 3521 | 2432 | 69.07 | 209 | 5.94 | 36 | 1.02 | 131 | 3.72 | 713 | 20.25 |
| **Henan** | 2791 | 2400 | 85.99 | 140 | 5.02 | 139 | 4.98 | 112 | 4.01 | 0 | 0 |
| **Hebei** | 2945 | 2352 | 79.86 | 15 | 0.51 | 368 | 12.50 | 211 | 7.16 | 0 | 0 |
| **Shaanxi** | 2426 | 2084 | 85.90 | 128 | 5.28 | 95 | 3.92 | 119 | 4.91 | 0 | 0 |
| **Fujian** | 2636 | 1551 | 58.84 | 292 | 11.08 | 122 | 4.63 | 19 | 0.72 | 652 | 24.73 |
| **Ningxia** | 1768 | 1415 | 80.03 | 22 | 1.24 | 194 | 10.97 | 136 | 7.69 | 0 | 0 |
| **Liaoning** | 2039 | 1410 | 69.15 | 57 | 2.80 | 194 | 9.51 | 51 | 2.50 | 327 | 16.04 |
| **Guizhou** | 2327 | 1354 | 58.19 | 831 | 35.71 | 97 | 4.17 | 45 | 1.93 | 0 | 0 |
| **Hubei** | 3037 | 1243 | 40.93 | 1647 | 54.23 | 82 | 2.70 | 65 | 2.14 | 0 | 0 |
| **Jiangxi** | 1477 | 1199 | 81.18 | 145 | 9.82 | 71 | 4.81 | 62 | 4.20 | 0 | 0 |
| **Guangxi** | 1939 | 1032 | 53.22 | 615 | 31.72 | 106 | 5.47 | 17 | 0.88 | 169 | 8.72 |
| **Gansu** | 1787 | 901 | 50.42 | 507 | 28.37 | 246 | 13.77 | 133 | 7.44 | 0 | 0 |
| **Heilongjiang** | 1111 | 895 | 80.56 | 32 | 2.88 | 141 | 12.69 | 43 | 3.87 | 0 | 0 |
| **Hunan** | 1552 | 849 | 54.70 | 574 | 36.98 | 99 | 6.38 | 30 | 1.93 | 0 | 0 |
| **Shanghai** | 864 | 835 | 96.64 | 0 | 0.00 | 19 | 2.20 | 10 | 1.16 | 0 | 0 |
| **Jilin** | 990 | 721 | 72.83 | 94 | 9.49 | 130 | 13.13 | 45 | 4.55 | 0 | 0 |
| **Tianjin** | 699 | 668 | 95.57 | 0.1 | 0.01 | 12 | 1.72 | 19 | 2.72 | 0 | 0 |
| **Chongqing** | 837 | 538 | 64.28 | 281 | 33.57 | 14 | 1.67 | 4 | 0.48 | 0 | 0 |
| **Sichuan** | 4167 | 513 | 12.31 | 3541 | 84.98 | 86 | 2.06 | 27 | 0.65 | 0 | 0 |
| **Beijing** | 456 | 434 | 95.18 | 11 | 2.41 | 4 | 0.88 | 6 | 1.32 | 0 | 0 |
| **Yunnan** | 3674 | 415 | 11.30 | 2960 | 80.57 | 250 | 6.80 | 50 | 1.36 | 0 | 0 |
| **Hainan** | 348 | 215 | 61.78 | 17 | 4.89 | 6 | 1.72 | 15 | 4.31 | 95 | 27.30 |
| **Qinghai** | 948 | 101 | 10.65 | 599 | 63.19 | 82 | 8.65 | 167 | 17.62 | 0 | 0 |
| **Tibet** | 87 | 1 | 1.15 | 70 | 80.46 | 0.2 | 0.23 | 14 | 16.09 | 1.8 | 2.07 |
| **Year** | **Total** | **Thermal power** | | **Hydroelectric** | | **Wind power** | | **Solar power** | | **Nuclear power** | |
|  |  | Production | Proportion (%) | Production | Proportion (%) | Production | Proportion (%) | Production | Proportion (%) | Production | Proportion (%) |
| **2020** | 76264 | 51770 | 67.9 | 13553 | 17.8 | 4665 | 6.1 | 2611 | 3.4 | 3665 | 4.8 |

**References**

1. National Bureau of Statistics. China Energy Statistical Yearbook (2020 Edition). Beijing: China Statistics Press; 2020.
